# Non‐Allergic Urticarial Skin Reactions Associated With MOv18 IgE, a First‐In‐Class IgE Antibody Recognising Folate Receptor Alpha

**DOI:** 10.1111/all.16514

**Published:** 2025-03-06

**Authors:** Chara Stavraka, Jitesh Chauhan, Silvia Crescioli, Sheila M. McSweeney, Amy Pope, Cheryl Gillett, Ashley Di Meo, Ioannis Prassas, Roman Laddach, Katie Stoker, Alexandra J. McCraw, Rebecca Adams, Thomas J. Tull, Nabeel Naban, Zena Willsmore, Kristina V. Semkova, Clive E. H. Grattan, John McGrath, Stephen J. Till, Christopher J. Corrigan, Rebecca Kristeleit, Sophia Tsoka, Eleftherios P. Diamandis, Katie E. Lacy, Sarah Pinder, Debra H. Josephs, James Spicer, Heather J. Bax, Sophia N. Karagiannis

**Affiliations:** ^1^ St. John's Institute of Dermatology, School of Basic & Medical Biosciences & KHP Centre for Translational Medicine King's College London London UK; ^2^ School of Cancer & Pharmaceutical Sciences King's College London, Guy's Hospital London UK; ^3^ King's Health Partners Cancer Biobank, School of Cancer & Pharmaceutical Sciences King's College London, Guy's Hospital London UK; ^4^ Department of Laboratory Medicine and Pathobiology University of Toronto Toronto Canada; ^5^ Division of Clinical Biochemistry Laboratory Medicine Program, Toronto General Hospital Toronto Canada; ^6^ Mount Sinai Hospital Toronto Canada; ^7^ Laboratory Medicine Program University Health Network Toronto Canada; ^8^ Department of Informatics, Faculty of Natural, Mathematical and Engineering Sciences King's College London London UK; ^9^ St. John's Institute of Dermatology Guy's and St Thomas' NHS Foundation Trust London UK; ^10^ Cancer Centre at Guy's Guy's and St Thomas' NHS Foundation Trust London UK; ^11^ King's Centre for Lung Health School of Immunology and Microbial Sciences, King's College London London UK; ^12^ Lunenfeld‐Tanenbaum Research Institute Mount Sinai Hospital Toronto Canada; ^13^ Department of Pathology and Laboratory Medicine Mount Sinai Hospital Toronto Canada; ^14^ Breast Cancer Now Research Unit, School of Cancer & Pharmaceutical Sciences King's College London, Guy's Cancer Centre London UK

**Keywords:** AllergoOncology, folate receptor alpha, IgE antibodies, transcriptomic analysis, urticarial skin reactions

## Abstract

**Background:**

IgE antibodies directed against cancer antigens have demonstrated potent anti‐tumour effects in pre‐clinical studies. MOv18 IgE, the first‐in‐class IgE recognising the cancer antigen folate receptor alpha (FRα), showed preliminary signs of efficacy in a Phase I trial. Treatment was well tolerated, with the most common adverse event being transient urticarial skin reactions. We investigated immunological and allergic response parameters associated with urticarial skin reactions in MOv18 IgE‐treated patients.

**Methods:**

Expression of target antigen, FRα, and MOv18 IgE reactivity with FRα or any component in human skin was studied by immunohistochemistry, immunofluorescence and immuno‐mass spectrometry. We conducted transcriptomic analyses in paired lesional and non‐lesional skin biopsies from a patient who developed an urticarial skin reaction. Systemic immunological markers including cytokines, β‐tryptase and basophil activation states were interrogated throughout the trial and contemporaneously with the skin reaction.

**Results:**

Of the 24 IgE‐treated patients, 62.5% developed transient urticarial skin reactions, with onset during the first infusion, diminishing with consecutive infusions and no β‐tryptase elevation nor clinical features indicating allergic aetiology. No FRα expression or MOv18 IgE binding to human skin was identified. Lesional skin biopsies from a patient given the highest antibody dose revealed scattered eosinophils, neutrophils and mast cell degranulation, but no increased immune cell infiltration. Transcriptomic analysis indicated pro‐inflammatory, but not allergic, pathway activation. No systemic allergic or hypersensitivity mediators or basophil activation were detected.

**Conclusions:**

Urticarial skin reactions following MOv18 IgE treatment were unlikely to result from allergic mechanisms or skin antigen recognition. The clinical presentation is consistent with infusion‐related reactions commonly observed with monoclonal antibody treatments.

**Trial Registration:**

EudraCT number: 2014‐000070‐19; ClinicalTrials.gov identifier: NCT02546921, registered 11/Sept/2015

## Graphical abstract

MOv18 IgE, the first‐in‐class IgE antibody targeting the cancer antigen folate receptor alpha (FR⍺) showed early signs of efficacy and was well tolerated in a first‐in‐human clinical trial, with transient urticarial skin reactions as the most common toxicity. This study, investigating the immunological parameters associated with these reactions, found no signs of skin antigen recognition by the antibody, no markers of allergic mechanisms in the patient blood or in the urticarial skin of a patient who received the highest antibody dose in the trial. These findings support the favourable safety profile of this novel anti‐cancer therapy class in the emerging field of AllergoOncology. ADAMTS9‐AS1, ADAMTS9 antisense RNA 1; CCL2, C–C motif chemokine ligand 2; FRα, folate receptor alpha; IHC, immunohistochemistry; IF, immunofluorescence; JUNB, JunB proto‐oncogene, AP‐1 transcription factor subunit; MOv18 IgE, the first‐in‐class IgE recognizing FRα; RNAseq, RNA sequencing; SELE, selectin E; TMA, tissue microarray.

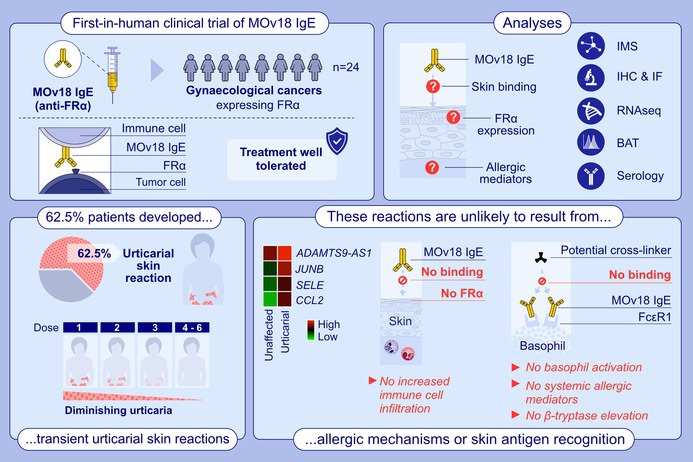

## 1 Introduction

Monoclonal antibodies (mAbs) have transformed the therapeutic landscape of haematological and solid malignancies [1]. Though all approved antibodies belong to the IgG class, interest in IgE class mAbs for tumour‐associated antigens (TAA) (AllergoOncology) is growing [2, 3, 4]. IgE antibodies recruit and activate potent effector cells and enhance immune surveillance [5]. MOv18 IgE is the first‐in‐class anti‐cancer IgE, recognising the human TAA, folate receptor alpha (FRα) [2], a glycosylphosphatidylinositol‐anchored membrane folate receptor that supports tumour growth [6]. FRα is overexpressed in up to 60% of epithelial ovarian tumours, and in subsets of endometrial, breast and lung cancers, but rarely in normal tissues. FRα is the target of the clinically validated IgG antibody‐drug conjugate, mirvetuximab soravtansine, approved for the treatment of ovarian cancers [7, 8, 9, 10, 11, 12, 13].

MOv18 IgE demonstrated promising anti‐tumour efficacy in several pre‐clinical models [5, 14, 15, 16, 17]. It became the first IgE antibody to be clinically evaluated in a Phase I trial in patients with solid tumours expressing FRα (NCT02546921), the results of which we recently reported [18]. MOv18 IgE showed early anti‐tumour activity and manageable toxicity; most adverse events (AEs) were low‐grade [≤grade 2 per NCI Common Terminology Criteria for Adverse Events (CTCAE)] [19]. The most common toxicity of MOv18 IgE was transient urticaria (with or without pruritus). A single patient experienced anaphylaxis, likely predicted by the detection of circulating basophils at baseline that could be activated by MOv18 IgE. The Basophil Activation Test (BAT) assay was used to avoid enrolling further patients with reactive basophils. The safety profile was deemed tolerable and the maximum tolerated dose has not been reached [18]. Most reactions occurred at the first or second exposure to MOv18 IgE, within the first hour of infusion, and diminished in severity upon repeated infusions. All urticarial rashes subsided within 24 h, either spontaneously or upon administration of systemic steroids and antihistamines [18].

This present study investigates skin AEs and potential immunological and allergic responses that may be associated with MOv18 IgE‐related cutaneous toxicity and evaluates these effects through the stratification of patients who experienced urticaria in comparison to those who did not. One mechanism may relate to the recognition of target antigen and stimulation of a potential allergic response in the skin. Thus, we examined FRα expression in human skin by immunohistochemistry (IHC) and evaluated MOv18 antibody clone reactivity with healthy donor human skin components by proteome‐wide immuno‐mass spectrometry (IMS). To investigate antigen recognition by MOv18 IgE and the potential for stimulation of an allergic pathway by cutaneous reactions, we used immunohistochemistry (IHC), immunofluorescence (IF) and transcriptomic analyses to compare paired lesional and non‐lesional skin biopsies from a patient who developed a CTCAE grade 3 urticarial reaction following treatment with MOv18 IgE at a 6 mg dose level (termed Patient A herein). Finally, we interrogated systemic immunological and allergy markers, including basophil activation, and the effect of intra‐patient dose escalation from 6 mg to 12 mg in Patient A, to understand if any of these parameters are associated with the urticarial reactions in MOv18 IgE‐treated patients.

## 2 Materials and Methods

### 2.1 Ethical Approval and Consent

Peripheral blood samples, cutaneous images and data were collected from patients (*n* = 24) in an open‐label, dose‐escalating Phase I study of MOv18 IgE for the treatment of advanced ovarian cancer, approved by the UK MHRA and NHS HRA. One patient, during the Phase I study, who developed a CTCAE grade 3 urticarial skin reaction (termed Patient A herein) had skin samples collected at Guy's and St. Thomas' NHS Trust under a study reviewed and approved by the Guy's REC (Reference 09/H0804/45). All patients gave written informed consent.

### 2.2 Adverse Events Reporting

NCI Common Terminology Criteria for Adverse Events (CTCAE) [Version 4] was used throughout for Adverse Event (AE) reporting and severity grading [19].

### 2.3 Evaluated Samples

**FIGURE 1:**
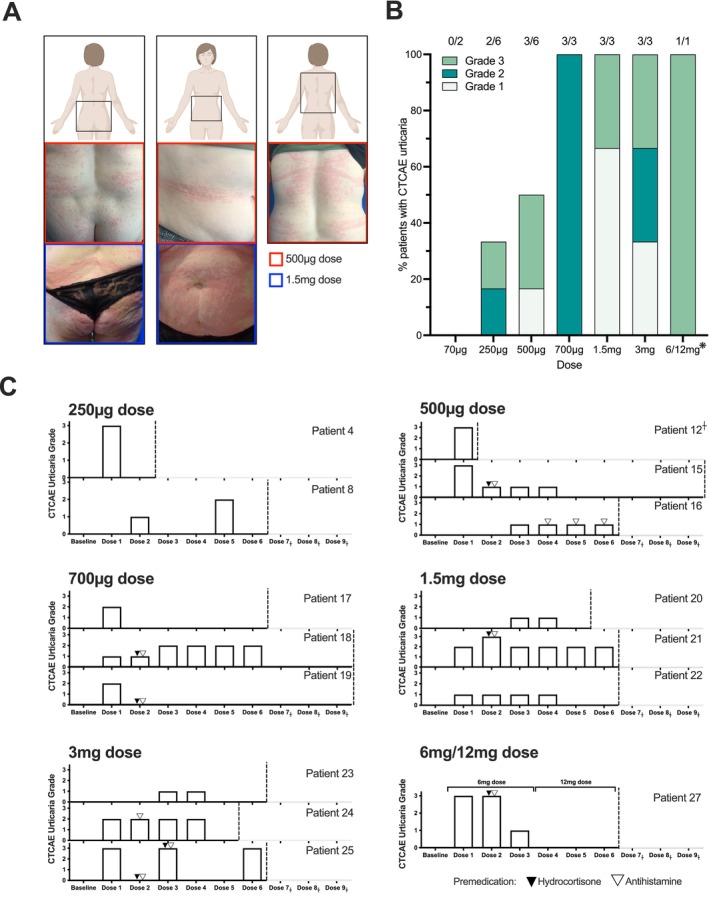
Readily manageable urticarial adverse events were associated with higher doses of MOv18 IgE treatment. (A) Panel of representative images of urticarial skin reactions, seen in a patient who received a 500 μg dose (purple) and a patient who received a 1.5 mg dose (orange) from the larger cohort of MOv18 IgE‐treated patients. (B) Proportion and CTCAE urticarial grade per dosing cohort of patients treated with MOv18 IgE (urticarial: *n* = 15; total *n* = 24). ^❋^Intra‐patient dose escalation was performed with this patient (*n* = 1) receiving 3 doses at 6 mg, followed by 3 doses at 12 mg. Number of patients treated, and number of patients who experienced urticaria, outlined per dosing cohort shown in Table S1. (C) Per dose CTCAE urticarial grade of patients that experienced urticarial symptoms (*n* = 15). The dotted line signifies when the patient stopped receiving MOv18 IgE treatment. Black and white arrows signify when the patient was given premedication with hydrocortisone and/or antihistamines, respectively.

**FIGURE 2:**
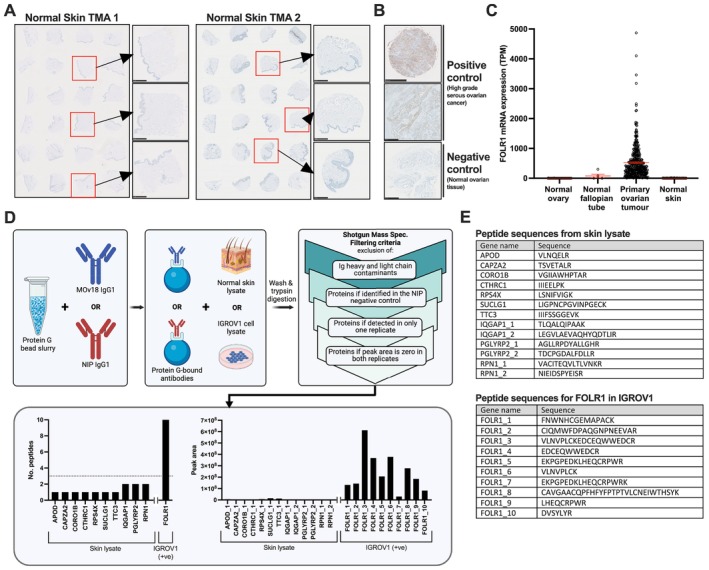
FRα is not expressed in the skin and immuno‐mass spectrometry (IMS) of MOv18 antibody clone pulldown demonstrated no skin‐associated binding. (A) Normal skin tissues stained with commercially available anti‐FRα antibody were negative for FRα staining (*n* = 48 across two TMAs). Further details of TMA sample map and demographic information included in Figure S1, Tables S2 and S3. (B) HGS ovarian tumour and normal ovarian control tissues were positively and negatively stained, respectively. (C) High FOLR1 mRNA expression in primary ovarian tumours (*n* = 419), but no or low expression in normal ovaries (*n* = 88), normal fallopian tube (*n* = 5) and normal skin (*n* = 556) (data extracted from Human Protein Atlas, v20.proteinatlas.org) [20, 21]. (D) Schematic of immuno‐mass spectrometry (IMS) experimental procedure and analysis pipeline for discovery of skin (healthy skin tissues lysate; *n* = 3) and IGROV1 cell lysate antigens, using MOv18 IgG and isotype control (NIP IgG) (upper row). Number of peptides found following pipeline filtering criteria in skin and IGROV1 lysates (dotted line signifying the cut‐off for which results are believed to be true, lower left). Peak area of peptides found in skin and IGROV1 lysates (lower right). Figure generated on BioRender. (E) Tables of peptide sequences observed for peptides identified in skin lysates (upper) and IGROV1 cell lysates (lower).

**FIGURE 3:**
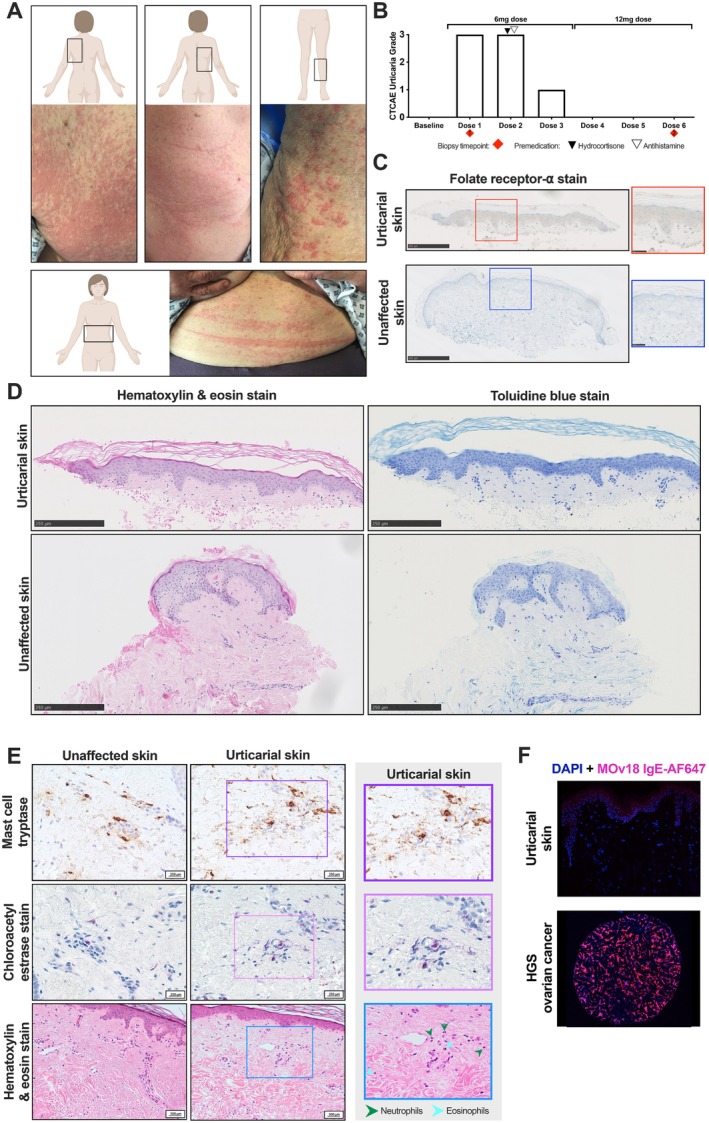
No evidence of FRα, immune cell infiltration or mast cell activation in urticarial and unaffected skin from a patient treated with the highest MOv18 IgE dose levels (Patient A). (A) Panel of images of the CTCAE grade 3 urticarial reaction observed in a patient during their first infusion of MOv18 IgE at 6 mg: Upper left back (upper left image), upper right back (upper centre image), left calf (upper right image) and abdomen (lower image). (B) Decreasing CTCAE urticarial grade was observed in a patient who experienced symptoms during treatment with MOv18 IgE. No urticarial symptoms were observed following intra‐dose escalation to a higher dose (12 mg). Red diamonds represent the biopsy collection timepoints for urticarial (timepoint 1) and unaffected (timepoint 2) skin samples analysed subsequently. (C) Staining for FRα in urticarial and unaffected skin indicated the absence of FRα in both tissues. Red and blue boxes correspond to respective zoomed‐in section of tissues. Biopsy timepoints as outlined in B. (D) Haematoxylin and eosin (H&E; left) and toluidine blue (right) staining show the absence of immune cell and mast cell infiltration, respectively, in comparison to unaffected tissue. Biopsy timepoints outlined in B. (E) Mast cell tryptase (MCT, upper row) and chloroacetyl esterase staining (CAE, middle row) shows evidence of enhanced mast cell degranulation in urticarial skin, which is not present in unaffected skin; higher magnification view of urticarial skin (right; purple and pink box, respectively). H&E staining (lower row) provides evidence of scattered neutrophils and eosinophils in urticarial skin (lower right) which is not present in unaffected skin; zoomed‐in section of urticarial tissue (right, blue box). (F) MOv18 IgE labelled with AlexaFluor647 (MOv18 IgE‐AF647, pink) did not bind to urticaria skin (upper) but did bind high‐grade serous ovarian tissue (lower).

**FIGURE 4:**
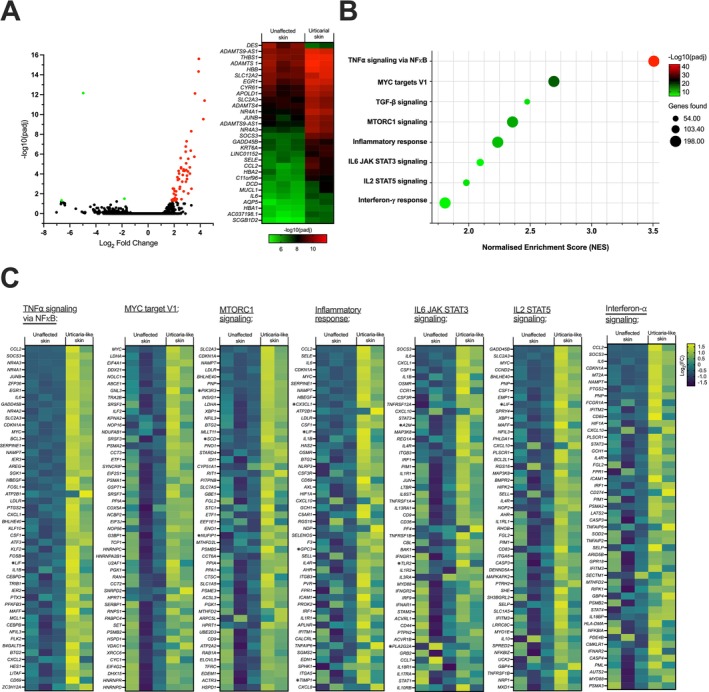
Gene expression and enriched pathways in urticarial tissue of a patient treated with the highest MOv18 IgE dose levels (Patient A). (A) Significantly differentially expressed genes were identified between urticarial and unaffected skin, using the limma package, ranked according to fold change. (B) Calculated enrichment of gene sets was evaluated within Hallmark, with selected example pathways shown, ranked according to fold change. (C) Top 50 most differentially expressed (FDR‐corrected) genes are shown for each selected pathway. ^❋^ *p* ≤ 0.05 for significant genes within the selected Hallmark pathways.

**FIGURE 5:**
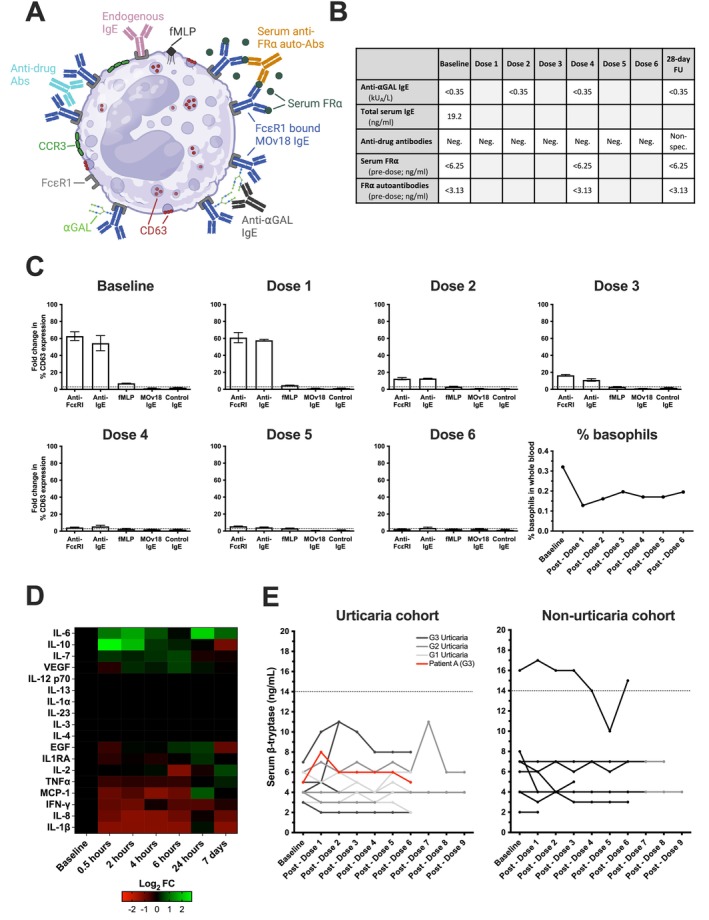
Diminishing CTCAE urticarial grade was associated with diminishing ex vivo basophil activation in a patient who experienced urticarial symptoms during MOv18 IgE treatment at the highest doses. (A) Overview of the potential mechanisms of basophil activation within the circulation: Anti‐drug antibodies (ADAs) could cross‐link FcɛRI‐bound MOv18 IgE; shed monovalent serum folate receptor alpha (FR) could form complexes with serum anti‐FR⍺ auto‐antibodies (auto‐Abs) and MOv18 IgE; and ⍺GAL (galactose‐alpha‐1,3‐galactose) found on the surface of SP2/0 produced MOv18 IgE may be cross‐linked by an anti‐⍺GAL IgE Abs. Figure generated on BioRender. (B) Table summarising levels of circulating mediators measured in Patient A. (C) In Patient A, no basophil activation (% CD63 expression) was triggered by MOv18 IgE or control IgE, however, diminishing IgE‐mediated (anti‐FcɛRI and anti‐IgE) ex vivo basophil activation was observed through treatment doses. The proportion of basophils in whole blood decreased from baseline levels following the first dose of MOv18 IgE, recovering slightly through subsequent doses (lower right). (D) Log_2_ foldchange (Log_2_FC) in median circulating cytokine levels post‐dose 1 (0.5, 2, 4, 6, 24 h and 7 days post‐dose) relative to normalised baseline values for Patient A. Overall increases in IL‐6, IL‐10, IL‐7 and VEGF were observed, with no change in IL‐12 p70, IL‐13, IL‐1⍺, IL‐23, IL‐3, IL‐4 and an overall decrease in EGF, IL1RA, IL‐2, TNF‐⍺, MCP‐1, IFN‐, IL‐8 and IL‐1β. (E) Serum β‐tryptase at baseline and throughout treatment in urticaria cohort (left; *n* = 14; colour denotes highest grade of CTCAE urticaria experienced by patient; red = Patient A who experienced grade 3 urticaria) and non‐urticaria (right; *n* = 9) patients. β‐tryptase levels in patients who experienced urticaria (urticaria cohort) (in the absence of anaphylaxis), consistently stayed below the upper limit of normal (14 ng/mL; dotted line) and no association with the grade of urticaria was observed. This is also consistent for patients who did not experience urticaria (non‐urticaria cohort); however, one patient was consistently above the upper limit of normal at baseline and through treatment—this was without any significant hypersensitivity or anaphylaxis experienced by this patient.

**FIGURE 6:**
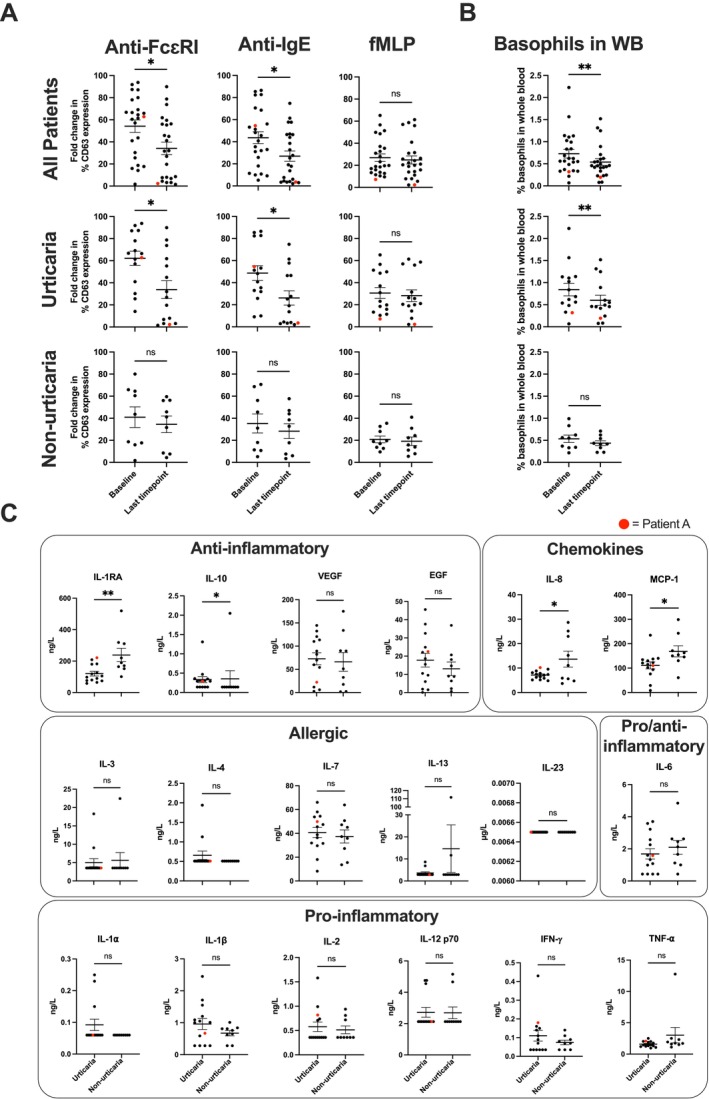
Reduced IgE‐mediated ex vivo basophil activation propensity and reduced circulating basophil levels basophils in patients who experienced urticarial symptoms through MOv18 IgE treatment. (A) Significantly decreased ex vivo basophil activation via IgE‐mediated mechanisms (anti‐FcɛRI and anti‐IgE) was observed in all patients (upper row) and in those patients who experienced any CTCAE grade urticaria following treatment with MOv18 IgE (middle row). Stimulation (% CD63 expression) was as measured via the basophil activation test at baseline and at the last available timepoint during MOv18 IgE treatment. No significant changes were observed in ex vivo basophil activation by a non‐IgE‐mediated (fMLP) stimulus in the whole patient cohort and in patients who experienced urticaria (upper and middle row: right). No significant changes in basophil activation were observed by IgE‐mediated and non‐IgE‐mediated stimuli in patients who did not experience urticaria through treatment with MOv18 IgE (lower row). All patients: *n* = 24, urticaria patients: *n* = 15, and non‐urticaria patients: *n* = 9. Patient A's datapoints highlighted in red. (B) Proportion of basophils in whole blood significantly significant decreased in all patients (upper, *n* = 24), and in those patients that experienced urticaria through treatment with MOv18 IgE (middle, *n* = 15). No significant changes in % of basophils in blood proportion were observed in those patients who did not experience any urticaria during treatment (lower, *n* = 9). Patient A's datapoints highlighted in red. (C) Circulating cytokine and chemokine levels at baseline, prior to treatment with MOv18 IgE, in urticaria (*n* = 14) and non‐urticaria (*n* = 9) patients. Urticaria patients show significant differences in anti‐inflammatory cytokines (IL‐1RA, IL‐10) and chemokines (IL‐8, MCP‐1). No differences in allergic or pro‐inflammatory cytokines. Patient A's datapoint highlighted in red. Data are shown as mean ± SEM. Wilcoxon matched‐pairs rank test (A: Upper and middle row; B: Upper and middle), paired *t*‐test (A: lower row, B: lower), unpaired *t*‐test (C: IL‐1RA, VEGF; EGF, IL‐8, MCP‐1, IL‐7, IL‐6, IL‐1α, IL‐1β, IFN‐α), Mann–Whitney test (C: IL‐10, IL‐3, IL‐4, IL‐13, IL‐23, IL‐2, IL12 p70, TNF‐α): *p ≤ 0.05, **p ≤ 0.01.

Figures 1, 5E, 6, Tables S1, S4–S6 illustrate data from 24 patients treated with MOv18 IgE in the first‐in‐man clinical trial, in relation to clinical, immunological and allergic response parameters associated with urticarial skin reactions. In Figure 2, we conducted (a) IHC of healthy volunteer normal skin tissue microarray samples (two microarrays of 24 core skin biopsies each); (b) transcriptomic analyses of FOLR1 mRNA expression in primary ovarian tumours (*n* = 419), normal ovaries (*n* = 88), normal fallopian tube (*n* = 5) and normal skin (*n* = 556) and (c) immuno‐mass spectrometry experiments with *n* = 3 healthy skin lysates. We obtained paired skin punch biopsies (*n* = 2 cores) from urticarial lesional and unaffected skin from the back of a patient with metastatic ovarian cancer treated with the highest MOv18 IgE dose and who developed a CTCAE grade 3 urticarial skin reaction (Patient A). With these we evaluated clinical and immunological features and signatures by IHC and transcriptomic analyses (Figures 3, 4; Figure S2); and associations of diminishing CTCAE urticarial grade in relation to several serum markers and basophil activation propensity and circulating levels (Figure 5B–D).

### 2.4 Studies in Human Samples

#### 2.4.1 Patient Skin Samples

Paired 4‐mm skin punch biopsies (*n* = 2 cores from *n* = 1 patient) were obtained from urticarial lesional and unaffected skin from the back of a patient with metastatic ovarian cancer treated with MOv18 IgE in the Phase I study who developed a CTCAE grade 3 urticarial skin reaction (Patient A).

#### 2.4.2 Tissue Microarrays (TMA)

Details on TMA and FRα expression evaluation by IHC are described in the Methods S1.

#### 2.4.3 Tissue IHC and IF

Toluidine blue staining and tissue IF of patient biopsy tissue are described in Methods S1.

#### 2.4.4 IMS

MOv18 IgG1, or negative control NIP IgG1, antibodies were tested for reactivity against tissue lysates from fresh frozen healthy skin (*n* = 3). IgG1 antibodies were loaded on protein G magnetic beads and incubated with lysates. The beads were washed, immunoprecipitated proteins digested with trypsin and analysed by liquid chromatography coupled to tandem mass spectrometry (LC–MS/MS) followed by parallel reaction monitoring mass spectrometry (PRM‐MS) (see Methods S1).

#### 2.4.5 Serological Investigations

Serum and whole blood samples were collected from Patient A and other MOv18 IgE‐treated patients at baseline and various timepoints during and after MOv18 IgE doses. Cytokine, anti‐drug antibodies (ADA), anti‐αGAL IgE antibodies, serum β‐tryptase, total serum IgE, anti‐FRα and autoantibodies detection, and Basophil Activation Test (BAT) were performed using blood drawn from these patients (see Methods S1).

### 2.5 Statistical Analyses

Data are presented as mean ± SEM. Analyses were performed on GraphPad Prism (v9.0). Appropriate statistical tests utilised were based on data distribution (which was evaluated with a Shapiro–Wilk test) and detailed in figure legends. Statistically significant differences are shown in the graphs (*p* values: *p ≤ 0.05, **p ≤ 0.01).

## 3 Results

### 3.1 The Frequency but Not the Severity of MOv18 IgE‐Induced Urticarial Skin Reactions Is Dose‐Dependent

In the Phase I trial, 24 patients with FRα‐expressing solid tumours received up to 6 weekly intravenous MOv18 IgE infusions, and some received 3 further maintenance doses every 2 weeks. Escalating doses from 70 μg to 12 mg were administered (Table S1). Approximately 62.5% of patients (*n* = 15) developed transient urticarial cutaneous reactions, with 25% (*n* = 6) developing CTCAE grade 3 urticaria (Figure 1A,B; Table S1) [19].

Most reactions occurred after the first or second exposure to MOv18 IgE and within the first hour of infusion initiation (Figure 1C). Reactions were transient urticated erythematous eruptions with or without pruritus, starting centrally and progressing to the limbs, accentuated at pressure‐prone areas and skin creases, with facial flushing in some cases. All urticarial reaction episodes resolved within 24 h, with or without systemic steroids and antihistamines, and without residual pigmentation. Some patients received pre‐medication with corticosteroids and/or antihistamines for subsequent doses following urticaria. The frequency of urticarial reactions increased at higher doses, but severity was patient dependent (Figure 1B). In most patients, reactions diminished with repeated doses (Figure 1C).

### 3.2 FRα Is Not Expressed in Normal Human Skin

It is possible that MOv18 IgE administration triggers a cutaneous reaction because FRα recognises an antigen in the skin. Therefore, we evaluated FRα expression in two normal skin TMAs by IHC using a commercially available anti‐FRα antibody and established protocols used to screen patients for inclusion in the MOv18 IgE trial (Figure 2A; Figure S1, Tables S2, S3) [18]. No FRα protein was detected in normal skin. Positive control high‐grade serous (HGS) ovarian cancer tissue expressed FRα, whereas normal ovarian tissue did not (Figure 2B). Transcriptomic analyses showed high *FOLR1* (FRα) expression in primary ovarian tumours, minimal expression in normal fallopian tubes and none in normal skin or non‐malignant ovarian tissues (Figure 2C).

### 3.3 Immuno‐Mass Spectrometry Identified No Antigen Recognition by the MOv18 Antibody Clone in Normal Human Skin Lysates

To evaluate if the MOv18 clone reacts with FRα or other antigens in skin, we performed proteome‐wide IMS [22, 23].

Positive controls included pooled normal skin lysates (*n* = 3) and FRα‐expressing human ovarian IGROV1 cancer cells. MOv18 IgG antibody, identical in variable regions to MOv18 IgE, was used due to the IgG specificity of the protocol [22]. Non‐binding hapten‐specific (NIP) IgG controlled for non‐specific staining. Antibodies immobilised to Protein G beads were incubated with either normal skin or IGROV1 cell lysates. IMS assessed pulleddown antigens filtered for non‐specific binding (Figure 2D, upper). MOv18 pulled down 10 possible skin antigens; however, none met minimum peptide hit and peak area criteria to be true targets (Figure 2D, lower). Contrastingly, IGROV1 cell lysate peptides corresponded to FRα (*FOLR1*), confirming MOv18 recognises FRα (Figure 2D, lower). Thus, the MOv18 clone has no detectable binding target in human skin.

Together, IHC and proteomic IMS data suggest that FRα is absent in human skin and the MOv18 clone does not bind any target in normal skin. These data exclude MOv18 IgE antigen recognition in the skin as a mechanism for the urticarial reactions.

### 3.4 Affected Skin Showed No Prominent Mast Cell Accumulation, but Evidence of Degranulation Consistent With a Urticarial Drug Reaction

We asked whether MOv18 IgE administration triggered immune cell influx in skin, which may explain the urticarial reactions. We studied the case of a 75‐year‐old patient with HGS ovarian cancer with a history of COPD and pulmonary embolism, but no allergies or infusion reactions, who received the highest dose levels of MOv18 IgE (3 weekly 6 mg infusions, escalated to 3 weekly 12 mg infusions) (Patient A). During the first two 6 mg infusions, the patient experienced a CTCAE grade 3 urticarial cutaneous reaction (Figure 3A) associated with flushing, shortness of breath and tachycardia, resolving completely within 24 h. She remained hemodynamically stable but required infusion interruption, slower rate re‐initiation and administration of supplemental nasal oxygen, intravenous fluids, systemic steroids and antihistamines. Prophylaxis was administered before subsequent infusions. The third 6 mg infusion caused a grade 1 urticarial skin reaction, with no urticarial reaction at 12 mg doses (Figure 3B).

A skin punch biopsy from a CTCAE grade 3 reaction area (back; first 6 mg dose) and a second biopsy from adjacent normal skin (back; collected upon final 12 mg dose, when no cutaneous reaction had occurred) demonstrated no FRα expression and the absence of immune cell infiltrates in the affected skin (Figure 3C,D). H&E staining revealed scattered eosinophils and neutrophils interstitially, consistent with a dermal hypersensitivity reaction (urticarial drug reaction; Figure 3E, lower row). There was no evidence of interface dermatitis or vasculitis in the examined tissues. Mast cell counts were similar in urticarial (15 per 5 HPF) and normal (14 per 5 HPF) skin (Figure 3D). MCT, CAE and CD117 staining confirmed low cutaneous mast cell numbers and showed no differences in mast cell numbers, but significant degranulation in urticarial skin (Figure 3E, upper and middle row; Figure S2) [24]. IF showed no MOv18 IgE binding in urticarial skin with fluorescently labelled MOv18 IgE‐AF647, except for non‐specific keratin layer staining, but positive control ovarian cancer tissue showed robust staining (Figure 3F).

In conclusion, no MOv18 IgE binding or prominent mast cell accumulation, but significant degranulation of mast cells and scattered eosinophil and neutrophil infiltrates, observed in urticarial skin from Patient A, indicates a urticarial drug reaction.

### 3.5 Transcriptomic Analysis of Affected Skin Reveals Activation of Pro‐Inflammatory Immune Pathways and Immune Signals Associated With Cutaneous Urticaria

To investigate pathways linked to MOv18 IgE‐induced urticarial reactions, gene expression analysis was performed on paired skin biopsies obtained from Patient A.

Differentially expressed genes, including *ADAMTS9‐AS1*, *JUNB*, *SELE* and *CCL2*, were identified between urticarial and normal skin (Figure 4A) [25, 26]. Gene set enrichment revealed several pro‐inflammatory immune pathways enriched in the urticarial tissue compared with normal skin (Figure 4B), including TNF‐α, MYC targets, MTORC1, inflammatory response and IL‐6, IL‐2, and IFN‐α signalling pathways (Figure 4C). These pathways are upregulated in chronic spontaneous urticaria [25, 26]. Overall, this indicates that MOv18 IgE triggers pro‐inflammatory and urticarial pathway activation at affected sites, consistent with clinical diagnosis.

### 3.6 Absence of Circulating Allergic Inflammation Markers or Hypersensitivity Signals Following MOv18 IgE Treatment

Without evidence of skin antigen recognition by MOv18 IgE (Figure 3E) or cutaneous allergic pathway stimulation (Figure 4), we investigated circulating allergic response markers that might explain the cutaneous reaction seen in Patient A.

We considered circulating immune mediators that may cross‐link MOv18 IgE on blood basophils and which could traffic to the skin, triggering degranulation and cutaneous reactions by forming IgE‐immune complexes on mast cells. We tested the serum for IgE antibodies to: galactose‐alpha‐1,3‐galactose (anti‐αGAL IgE), a carbohydrate found on MOv18 IgE; ADAs (anti‐MOv18 IgE); soluble FRα antigen and anti‐FRα auto‐antibodies, which have the potential to cross‐link MOv18 IgE (Figure 5A). No evidence of these potential cross‐linkers was found before, during or after treatment. Therefore, no known complex‐forming immune cell activators were detected (Figure 5B).

The BAT was used to interrogate basophil stimulation capacity throughout treatment and successfully predicted hypersensitivity to the therapeutic agent [18, 27]. BAT on blood from Patient A showed no basophil activation by MOv18 IgE or control IgE (Figure 5C). In Patient A, after the first dose, reduced basophil activation propensity ex vivo by IgE‐mediated (anti‐FcɛRI and anti‐IgE) stimuli was observed with repeated MOv18 IgE treatment (Figure 5C). Alongside, a diminishing cutaneous reaction was observed over the course of treatment in this patient (Figure 3B). Basophil activation ex vivo in response to fMLP (non‐IgE‐mediated activation), albeit low at baseline, remained unaffected throughout treatment. Additionally, during treatment with MOv18 IgE, there was a reduction in the percentage of circulating basophils. This decrease was followed by a partial recovery and then stable basophil levels during subsequent doses (Figure 5C).

Furthermore, cytokine analysis of sera from Patient A showed unchanged levels of allergic mediators IL‐4 and IL‐13, but elevated anti‐inflammatory IL‐10 and pro‐inflammatory IL‐6 cytokines within 2 h following infusion, coinciding with the cutaneous reaction. Pro‐inflammatory mediators IFN‐γ, IL‐8, IL‐1β and MCP‐1 decreased within 2 h following the MOv18 IgE dose (Figure 5D). Together, the lack of basophil stimulation and no elevated levels of allergic mediators indicate no systemic hypersensitivity to MOv18 IgE for this patient. In concordance, we found no clinical signs of anaphylaxis or elevation of serum β‐tryptase (< 14 ng/mL) in Patient A, as well as the whole cohort, irrespective of urticaria. No MOv18 IgE‐treated patient who experienced urticaria had abnormally elevated serum β‐tryptase (Figure 5E).

In the MOv18 IgE treatment cohort, a significant decrease in IgE‐mediated basophil activation propensity ex vivo was observed through treatment when compared to baseline values in those patients who experienced urticaria (Figure 6A, upper and middle rows). This decrease was not seen in patients who did not experience urticaria (Figure 6A, lower row). Similarly, a significant reduction in the proportion of circulating basophils following treatment was observed in MOv18 IgE‐treated patients who experienced urticaria during treatment (Figure 6B, left and middle). This was not seen in those patients who did not experience urticaria (Figure 6B, right). Additionally, no differences were seen in circulating allergic or pro‐inflammatory cytokines at baseline prior to IgE treatment in patients who subsequently experienced urticaria compared to those who did not. Lower serum levels of anti‐inflammatory IL‐1RA, and chemokines IL‐8 and MCP‐1, and higher levels of anti‐inflammatory IL‐10, were detected at baseline prior to treatment in those patients who subsequently experienced urticaria (Figure 6C).

Analysis of potential circulating mediators of basophil activation [anti‐drug antibodies (ADA), basophil activation by MOv18 IgE ex vivo, soluble FRα, autoantibodies to FRα, total IgE and anti‐αGAL IgE] did not identify a possible association of markers in patients who experienced urticaria at any grade without systemic anaphylaxis (Table S4) versus those who did not experience urticaria (Table S5). This differs from the single patient who experienced anaphylaxis during the MOv18 IgE clinical trial, who also experienced CTCAE grade 3 urticaria but additionally had a positive BAT result (> 3‐fold change increase in CD63 expression on basophils) when their blood was incubated with MOv18 IgE ex vivo (Table S6).

In summary, repeated dosing of MOv18 IgE in Patient A and in other patients who experienced urticaria during treatment was associated with reduced IgE‐mediated basophil activation propensity and lower levels of circulating basophils. No circulating allergic inflammatory or hypersensitivity mediators were detected that could account for the reactions in Patient A and in other patients who experienced urticaria following treatment in comparison to those who did not experience urticaria.

## 4 Discussion

MOv18 IgE had a safety profile that was tolerable in the Phase I trial, and the maximum tolerated dose has not been achieved. The primary adverse event observed in the Phase I trial was transient urticaria. We sought to investigate several factors that might explain the transient urticarial reactions observed during MOv18 IgE treatment in the Phase I clinical trial.

We examined cutaneous reaction prevalence and severity in MOv18 IgE‐treated patients. Urticarial reactions of any grade, with or without mild systemic symptoms, were observed in 62.5% of the patients, with those treated at higher doses more likely to experience symptoms but no clear relationship between symptom severity and dose level (Figure 1). Most reactions occurred during the initial MOv18 IgE doses, diminished with subsequent infusions and/or with prophylactic medications, but were without serum β‐tryptase elevation in the blood, or signs of basophil activation by MOv18 IgE ex vivo. These clinical and biological features align with standard infusion‐related reactions commonly observed in (up to 77%) patients receiving IgG mAbs [28, 29].

We investigated if MOv18 IgE triggers a cutaneous reaction by recognising a skin antigen and inducing mast cell degranulation in the affected site. IHC (Figures 2 and 3) and transcriptomic analyses (Figure 4) [20, 21], revealed no evidence of FRα expression in normal skin or in paired urticarial and normal skin biopsies from a MOv18 IgE‐treated patient (Patient A). Using IF and IMS, we found no binding of MOv18 IgE to human urticarial or normal skin (Figures 2 and 3). These collectively exclude direct recognition of FRα or any skin antigens that could trigger MOv18 IgE to cross‐link FcεRI‐expressing immune cells (i.e., mast cells, basophils).

In the case study of Patient A, treated with the highest doses of MOv18 IgE (initially 6 mg, escalated to 12 mg), we investigated the mechanism of CTCAE grade 3 urticarial reaction by examining cutaneous and systemic immunological and allergic response markers. We found no increased immune cell infiltration in affected skin compared to normal skin (by toluidine blue, CAE, CD117 and H&E), although mast cell degranulation (MCT and CAE) was evident within urticarial skin [24]. Scattered neutrophils and eosinophils were noted; however, granulocytes present at the onset of urticaria may have been ablated following stimulation sand degranulation, making their detection challenging. It is unlikely that direct antigen recognition and subsequent degranulation by MOv18 IgE‐FcεR complexes on skin‐resident immune cells drive these urticarial reactions in this patient. However, it is possible that immune cells and circulating mediators may contribute to these cutaneous manifestations.

Transcriptomic analysis of the skin biopsies from Patient A revealed several DEGs, including *SELE*, *CCL2*, *ADAMTS9* and *JUNB*, known to be involved in urticarial pathways [25, 26]. Hallmark pathway analysis indicated enrichment of pro‐inflammatory immune pathways, whereas allergic response pathways were not active. The gene expression profiles in the urticarial skin sample suggest an urticaria phenotype. Given the absence of FRα expression in skin and the lack of allergic response pathways, it is unlikely that local multivalent antigen binding and cross‐linking IgE‐FcεRI complexes on mast cells or recruited immune cell activation drive these reactions.

Triggering systemic IgE‐mediated reactions is the main perceived risk of IgE therapeutic agents. A cutaneous reaction could be triggered by the formation of IgE immune complexes on FcεRI‐expressing basophils, leading to their activation and degranulation. We sought to study several potential circulating cross‐linkers that may cause urticarial reactions in patients, but no or negligible levels of anti‐αGAL IgE, FRα and anti‐FRα autoantibody immune complexes, high serum IgE levels and ADAs were found (Figure 5). In patients who experienced urticaria, lower serum levels of IL‐1RA, which exerts antagonistic activity to IL‐1β may suggest a predisposition to mast cell activation in the skin through the IL‐1β pathway, as well as point to skewed cytokine and chemokine profiles, which might influence susceptibility to urticarial reactions to IgE immunotherapy (Figure 6) [30, 31].

We also utilised the BAT, a standard allergy assay to assess the potential for drugs to induce type I hypersensitivity, which has been increasingly applied in cancer [27, 32, 33, 34, 35]. In the Phase I trial, we showed that prior to the administration of MOv18 IgE, the BAT predicted the anaphylaxis experienced by one patient and can now be used to exclude patients in future trials that have the potential to develop type I hypersensitivity [18]. We and others have used the assay to confirm type I hypersensitivity to chemotherapy agents and therapeutic antibodies [30], like cetuximab [32, 34, 35, 36]. Despite the onset of urticarial reactions, we observed consistently no response of the patient's basophils to ex vivo stimulation with MOv18 IgE in the BAT, at baseline and throughout treatment. This suggests that the urticarial responses are unlikely to be linked to blood basophil activation and are likely to have a different mechanism of action compared to the single patient who experienced anaphylaxis. This is supported by consistent normal‐range serum β‐tryptase levels in all patients, including those who experienced urticarial skin reactions. However, diminishing basophil propensity for activation by immune stimulation ex vivo was seen in patient blood samples following subsequent MOv18 IgE infusions. This was more prominent with patients who experienced urticaria and only via IgE‐mediated immune stimuli (Figures 3 and 6). Based on these observations, it could be hypothesised that repeated IgE treatment results in partial ablation, sequestering or exhaustion of basophil populations. Understanding this phenomenon would require further investigation, including additional studies into the lifespan of basophils in human circulation, which is not fully elucidated [37]. Pre‐medication with corticosteroids and antihistamines was only utilised prior to the administration of dose 2 and is unlikely to have driven these reductions in urticarial reaction severity (Figure 3B).

We examined whether MOv18 IgE treatment and the transient urticarial reactions were linked with altered serum cytokine levels in Patient A. The observed increase in serum IL‐6 and IL‐10 is consistent with our findings in the blood of other MOv18 IgE Phase I trial patients [18] and consistent with the cytokine profiles reported in the literature in acute urticaria [38]. No elevation of IL‐4, a cytokine implicated in allergy, was detected in the 24 h following treatment in Patient A, nor in any other patient treated with MOv18 IgE [18]. These results were consistent with the absence of IL‐4 signalling and enhanced pro‐inflammatory (e.g., TNF‐α, IL‐6, IL‐2, IFN‐α) signalling pathways seen in transcriptomic analysis of the urticarial skin tissues. Further investigation is necessitated to understand if altered cytokines are induced by, or resulting from, urticarial skin reactions, and if these contribute to the urticaria in these MOv18 IgE‐treated patients.

The observed clinical presentation (onset at the first and/or second infusion, and diminishing over subsequent treatments), the complete resolution with supportive medication (including systemic steroids and anti‐histamines), alongside the cytokine profiles identified, and the exclusion of type I hypersensitivity markers corroborate a typical diagnosis of an infusion‐related reaction commonly described with the treatment of IgG biologics in oncology [29, 39, 40].

## 5 Conclusion

We investigated potential immunological and allergic response parameters that may underpin the urticarial skin reactions observed in patients treated within the first‐in‐man Phase I clinical trial of an anti‐cancer IgE therapeutic. We found no evidence of an IgE‐mediated allergic response in the skin of a patient treated with the highest dose of MOv18 IgE. Instead, we observed, in the urticarial skin of this patient, an upregulation of urticarial and pro‐inflammatory signalling pathways, suggesting a resemblance to infusion‐related reactions commonly seen with IgG mAbs. These reactions can be effectively managed during treatment. Further research may uncover yet undefined systemic and/or local mediators that drive these transient urticarial responses. Our data support the favourable safety profile of this novel anti‐cancer therapy in the emerging field of AllergoOncology.

## Ethics Statement

The clinical study was approved by the UK Medicines and Healthcare products Regulatory Agency and the National Health Service Health Research Authority (London Bridge Research Ethics Committee) (EudraCT number 2014‐000070‐19; ClinicalTrials.gov identifier NCT02546921, first registered 11 September 2015). Skin samples were collected at Guy's and St. Thomas' NHS Trust under a study reviewed and approved by the Guy's REC (Reference 09/H0804/45). Participants provided written informed consent prior to the conduct of any study procedures.

## Conflicts of Interest

J.S. and S.N.K. are founders and shareholders of Epsilogen Ltd. H.J.B. is employed through a fund provided by Epsilogen Ltd. J.C. has been employed through a fund provided by Epsilogen Ltd. S.N.K., J.S., D.H.J. and H.J.B. declare patents on antibodies for cancer. R.K. is the Lead Investigator for the Phase IB/II MOv18 trial and has received travel support from Epsilogen Ltd. All other authors declare no conflicts of interest.

## Supporting information


Data S1.

## Data Availability

The data that support the findings of this study are available from the corresponding author upon reasonable request.
