# Supplementary material for: Non‐Allergic Urticarial Skin Reactions Associated With MOv18 IgE, a First‐In‐Class IgE Antibody Recognising Folate Receptor Alpha
Source: Allergy. 2025 Mar 6;80(8):2225–39. doi: 10.1111/all.16514 (PMC12368751; doi:10.1111/all.16514)
Supplement: Supplementary file 1 — Data S1. [file ALL-80-2225-s001.docx]

**Supplementary Materials**

Supplementary Methods

**Studies in healthy human samples**

**Tissue microarrays (TMA):** Two normal skin tissue formalin fixed paraffin embedded (FFPE) tissue microarrays (TMAs) (catalogue number #SK244 and #SK244a, each TMA including 12 cases in duplicate cores) and an ovarian cancer frozen TMA (#FOV401a, 20 cases, duplicate cores) were obtained from US Biomax Inc (Rockville, MD). Demographic and further histopathological details for these TMAs can be found at: <http://www.biomax.us/>.

**Studies in samples from Patient A**

**Evaluation of FRα expression by immunohistochemistry (IHC):** IHC was used to evaluate the membrane and cytoplasmic expression of FRα in normal skin tissue microarrays (TMAs) as well as the patient derived paired skin biopsies. Novocastra^TM^ Liquid mouse anti-human FRα primary antibody (Leica) was applied to FFPE sections for 32 minutes at room temperature at 1/500 dilution, followed by detection with Ultra Universal 3,3'-diaminobenzidine (DAB) detection kit (Ventana Medical Systems Inc.) and then Haematoxylin II applied for 8 minutes. Scoring of tissue sections was performed by a consultant Pathologist, with FRα positivity determined as ≥5% staining at any intensity.

**Toluidine blue stain:** Toluidine blue staining was used to evaluate the presence of tissue mast cells in patient-derived affected urticarial and unaffected skin samples. Tissue sections were deparaffinized and hydrated with distilled water. Toluidine blue working solution (pH 2.0-2.5) was applied for 2-3 minutes, and the sections were then washed 3 times with distilled water. Dehydration was performed quickly with 95% and 2 changes of 100% alcohol and the sections were then cleared in xylene for 2 changes, 3 minutes each. Mast cells were counted by a consultant Pathologist.

**Tissue Immunofluorescence (IF):** Half of the 4mm skin punch biopsy was snap frozen in liquid nitrogen and used for IF. MOv18 IgE was directly labelled with Alexa Fluor 647 fluorophore using the Alexa Fluor™ 647 Antibody Labelling Kit (ThermoFisher Scientific). Frozen tissues were thawed at room temperature for 10 minutes, allowed to dry and fixed with 4% paraformaldehyde (PFA) for 10 minutes. Once PFA was removed, tissues were washed 3 times with PBS. Sections were covered with Human AB serum (Sigma) for 1 hour at room temperature. Tissues were incubated overnight with MOv18 IgE-AF647 1:300 concentration and kept at 4°C in the dark. Sections were washed 3 times in PBS and allowed to dry at RT. ProLong™ Gold Antifade Mountant with DAPI (ThermoFisher Scientific) was added, coverslips were applied, and slides solidified overnight at room temperature in the dark. Stained sections images were acquired using Nikon A1 Inverted Confocal microscope.

**Immuno-mass spectrometry (IMS)**

Pellets containing 20 to 50x10^6^ target expressing IGROV1 cells (stage III ovarian solid tumour origin; female 47 years), or tissue lysates generated from three pooled human skin samples, were resuspended in 1.75ml lysis buffer (PBS, 0.1% Tween20, 1X Halt™ protease inhibitors cocktail - Thermo Scientific) in a 15mL tube, incubated at 4°C on a roller and vortexed for 20 minutes. 300µL ProteinA Dynabeads® (Invitrogen) were prepared with 100µg MOv18 humanized IgG1 in 800uL Binding Buffer as per manufacturer's protocol. Cell lysates were centrifuged for 10 minutes at 4500rcf at 4°C and supernatants were transferred to the washed MOv18 Dynabeads®. Beads with immunoprecipitated fraction were placed in 30uL of Elution Buffer and 10µL of 4X LDS sample buffer (Invitrogen) and stored at -20°C. Samples were thawed, beads were concentrated on a magnet, supernatant was transferred to a 1.5mL microcentrifuge tube and β-mercapto-Ethanol was added to a final concentration of 5% (v/v). Samples were incubated for 10 minutes at 95°C before being resolved on a 4-12% gradient NuPAGE™ gel (Invitrogen) at 200 Volts in MOPS buffer. Migration was stopped when Coomassie G250 reached the bottom of the gel. The gel was fixed in 7% Acetic Acid/40% methanol (v/v) for 30 minutes at room temperature and stained with a 1X solution of colloidal Brilliant Blue G with 20% methanol for 1 hour at room temperature. The gel was first destained for 5 minutes with 7% Acetic Acid and 25% methanol and then overnight with 2% acetic Acid and 25% methanol and stored in distilled water. Bands of interest were cut out and sent to Aulesa Biosciences for mass spectrometry analysis.

Proteins in gel slices were reduced (DTT), alkylated (iodoacetamide) and digested overnight with trypsin. Peptides within the tryptic digests were fractionated using an Ultimate 3000 nano-LC system in line with an Orbitrap Fusion Tribrid mass spectrometer (ThermoFisher Scientific). In brief, peptides in 1% (v/v) formic acid were injected onto an Acclaim PepMap C18 nano-trap column (ThermoFisher Scientific). After washing with 0.5% (v/v) acetonitrile 0.1% (v/v) formic acid peptides were resolved on a 250 mm x 75 μm Acclaim PepMap C18 reverse phase analytical column (ThermoFisher Scientific) over 150 min using 7 gradient segments (1-6% solvent B over 1min, 6-15% B over 58min, 15-32%B over 58min, 32- 40%B over 5min, 40-90%B over 1min, held at 90%B for 6min and then reduced to 1%B over 1min) with a flow rate of 300 nL.min−1 . Solvent A was 0.1% formic acid, and Solvent B was aqueous 80% acetonitrile in 0.1% formic acid. Peptides were ionized by nano-electrospray ionization at 2.2 kV using a stainless-steel emitter with an internal diameter of 30 μm (ThermoFisher Scientific) and a capillary temperature of 250°C. All spectra were acquired using an Orbitrap Fusion Tribrid mass spectrometer controlled by Xcalibur 2.0 software (ThermoFisher Scientific) and operated in data-dependent acquisition mode. FTMS1 spectra were collected at a resolution of 120 000 over a scan range (m/z) of 350-1550, with an automatic gain control (AGC) target of 400 000 and a max injection time of 100ms. The Data Dependent mode was set to Cycle Time with 3s between master scans. Precursors were filtered according to charge state (to include charge states 2-7), with monoisotopic precursor selection and using an intensity range of 5E3 to 1E20. Previously interrogated precursors were excluded using a dynamic window (40s +/-10ppm). The MS2 precursors were isolated with a quadruple mass filter set to a width of 1.6m/z. ITMS2 spectra were collected with an AGC target of 5000, max injection time of 50ms and HCD collision energy of 35%. LC-MS/MS data was processed using Proteome Discoverer (ThermoFisher Scientific) with database searching against a downloaded FASTA file originating from Uniprot_SwissProt_2019_02. Results were initially visualized within the software and then exported to Excel for further review. The list of proteins obtained was filtered by removing Ig heavy and light chains and protein contaminants (keratins, complement, apolipoproteins); proteins identified in the anti-NIP negative control; and proteins identified in only one of the two technical replicates, as previously described^1-3^.

**Serological investigations**

Serum samples were collected from the same patient who underwent paired skin punch biopsies at baseline (Patient A; intrapatient dose escalation - 6mg/12mg dose), and from *n*=23 who also received treatment with MOv18 IgE (250µg to 3mg dose, dependent on cohort) and at several timepoints during and after the administration of the consequent MOv18 IgE doses for the following investigations:

**Cytokine assay:** Multiplex analysis of a cytokine panel relevant to IgE biology^4-7^ was performed using the Randox cytokine arrays, as follows, at baseline and at several timepoints following the first MOv18 IgE dose. Randox Custom Cytokine Array I (Normal or high sensitivity): Epidermal Growth Factor (EGF), Interferon-γ (IFN-γ), Interleukin-1α (IL-1α), Interleukin-1β (IL-1β), Interleukin-2 (IL-2), Interleukin-4 (IL-4), Interleukin-6 (IL-6), Interleukin-8 (IL-8), Interleukin-10 (IL-10), Monocyte Chemotactic Protein-1 (MCP-1), Tumour Necrosis Factor-α (TNF-α), Vascular Endothelial Growth Factor (VEGF), and Interleukin-1 receptor alpha (IL-1r⍺). Randox Cytokine Array V: Cytokine Array V, Interleukin-3 (IL-3), Interleukin-7 (IL-7), Interleukin-13 (IL-13), Interleukin-12 p70 (IL-12 p70), and Interleukin-23 (IL-23)

**Anti-drug antibodies (ADA):** Anti-drug antibody (ADA) samples were drawn at baseline and 2- and 5-weeks following initiation of weekly dosing. Additional samples were collected 28 days after the last dose was administered and following any suspected infusion reaction. Serum samples were stored frozen at -80°C prior to analysis using an experimental qualitative bridging ELISA assay with MOV18 IgE to detect the presence of ADA’s, as previously published^8^. In brief, the assay involves a screening assay where incubating standards and patient serum samples are loaded in a microplate that has been pre-coated with MOv18. Any anti-drug antibody present in the sample will bind to the immobilised antibody. After washing away any unbound substances, biotinylated MOv18 is added to the wells. A streptavidin-horseradish peroxidase (HRP) conjugate is then added, and enzymatic activity is measured as an optical density at 492nm using o-phenylenediamine dihydrochloride (OPD) as a substrate. A second confirmatory assay was then performed on all samples that had measurable levels of binding in the first screening ELISA. In this second confirmatory ELISA, sera were pre-incubating with MOv18, then assessed as described above. Goat anti-human IgE was used as a surrogate positive control, and pooled normal human serum as a negative control. Classification of results: “negative” = no measurable binding in screening ELISA; “non-specific” = measurable binding in screening and confirmatory ELISA; and “positive” = measurable binding in screening ELISA and no measurable binding in confirmatory ELISA.

**Anti-⍺GAL (galactose-α-1,3-galactose) IgE antibodies:** Anti-⍺GAL IgE antibodies were measured using commercially available routine ImmunoCAP test system with Phadia 250 instrument (Thermo Fisher Scientific) in patient sera before, during and after MOv18 IgE treatment.^8^ Throughout treatment, samples from three patients were measured to have ⍺GAL IgE antibody titres >0.1 kUA/L (threshold of positivity according to reference laboratory). Grade interpretation (kUA/L): Grade 0 = ≤0.35; Grade 1 = 0.36-0.70; Grade 2 = 0.71-3.50; Grade 3 = 3.51-17.50; Grade 4 = 17.51-52.50; Grade 5 = 52.51-100.00; and Grade 6 = 100.01-999.99.

**Serum β-Tryptase:** No features of anaphylaxis or other manifestation of allergic toxicity were observed in preclinical animal models, but in addition to the risk mitigation steps described above serial measurements of serum β-tryptase were included in this trial. Β-tryptase is released by degranulation of activated effector cells, and elevation is characteristic of anaphylaxis.^9^ The protocol mandated β-tryptase measurement at baseline, at the end of each infusion, and following any infusion-related event, primarily to distinguish cytokine release from anaphylaxis. It was also measured following the onset of urticaria, which can occur as a feature of anaphylaxis.^10^ β-tryptase was measure by ELISA as part of clinical assays undertaken in Addenbrookes Hospital, Cambridge, U.K.

**Total Serum IgE:** Blood samples for serum IgE evaluation were taken at baseline, prior to MOV18 IgE treatment, and analysed using commercially available ImmunoCap IgE test with Phadia 2500 automated instrument for diagnostic purposes. Lower limit of quantification: <4.8 ng/ml**.** Upper limit of normal serum IgE = 81ng/ml**.**

**Anti-FRα and autoantibodies ELISAs:** Further blood samples were drawn at baseline, 3 weeks following initiation of weekly dosing and 28 days after the last dose, as well as following any observed adverse events. Serum was separated and stored at -80^o^C prior to evaluation of circulating soluble FRα and anti-FRα antibodies by ELISA, as previously described.^11^ Briefly, FRα in patient sera was captured and detected by specific antibodies, the detection antibody being conjugated with biotin for development with streptavidin. Similarly, anti-FRα antibodies were captured by recombinant FRα, and detected by specific IgG1 antibody and anti-IgG1-HRP. Levels were quantified by recombinant FRα and anti-FRα IgG1 standard curves, with lower levels of quantification (LLOQ) of 6.25 ng/ml and 3.125 ng/ml in serum, respectively.

**Basophil Activation Test (BAT):** Basophil activation tests (BAT) were performed as previously described^12,13^ on fresh whole blood from this urticarial sample patient (Patient A) using aliquots of the trial supply of MOv18 IgE, and a laboratory standard solution. Briefly, basophils in unfractionated whole blood samples were incubated with positive controls (anti-FcεRI, fMLP and anti-IgE) or MOv18 and control IgE antibodies for 30 minutes at 37°C. Staining antibodies were used for CCR3 (to identify basophils) and CD63 (as a marker of activation). Data were analysed as the fold change in percentage CD63 expression relative to the patient’s background. BAT was performed at baseline and after administration of each intravenous dose.

**Transcriptomic analysis of urticarial lesional and unaffected skin**

Half of the 4mm skin punch biopsy from Patient A was formalin fixed and paraffin embedded. RNA extraction, library preparations, and sequencing reactions were conducted at Azenta (South Plainfield, NJ, USA). Total RNA was extracted using Qiagen RNeasy FFPE kit following manufacturer’s instructions (Qiagen).

RNA samples were quantified using Qubit 2.0 Fluorometer (ThermoFisher Scientific) and RNA integrity was checked with 4200 TapeStation (Agilent Technologies).

rRNA depletion sequencing library was prepared by using QIAGEN FastSelect rRNA HMR Kit (Qiagen). RNA sequencing library preparation uses NEBNext Ultra II RNA Library Preparation Kit for Illumina by following the manufacturer’s recommendations (NEB). Briefly, enriched RNAs are fragmented for 15 minutes at 94°C. First strand and second strand cDNA are subsequently synthesized. cDNA fragments are end repaired and adenylated at 3’ends, and universal adapters are ligated to cDNA fragments, followed by index addition and library enrichment with limited cycle PCR.

Sequencing libraries were validated using the Agilent Tapestation 4200 (Agilent Technologies) and quantified using Qubit 2.0 Fluorometer (ThermoFisher Scientific) as well as by quantitative PCR (KAPA Biosystems). The sequencing libraries were multiplexed and clustered on one flowcell. After clustering, the flowcell was loaded on the Illumina HiSeq instrument according to manufacturer’s instructions. The samples were sequenced using a 2x150 Pair-End (PE) configuration. One mismatch was allowed for index sequence identification.

After demultiplexing, sequence data was checked for overall quality and yield. Then, sequence reads were trimmed to remove possible adapter sequences and nucleotides with poor quality using Trimmomatic v.0.36. The trimmed reads were mapped to the reference genomes using the STAR aligner v.2.5.2b. Unique gene hit counts were calculated by using feature Counts from the Subread package v.1.5.2 Only unique reads within exon regions were counted.

After extraction of gene hit counts, the gene hit counts table was used for downstream differential expression analysis. Using DESeq2, a comparison of gene expression between the groups of samples was performed. The Wald test was used to generate p-values and Log2 fold changes. Genes with adjusted p-values <0.05 and absolute log2 fold changes >1 were called as differentially expressed genes for each comparison. A gene ontology analysis was performed on the statistically significant set of genes by implementing the software GeneSCF v1.1. The GO list was used to cluster the set of genes based on their biological process and determine their statistical significance. A PCA analysis was performed using the "plotPCA" function within the DESeq2 R package. The plot shows the samples in a 2D plane spanned by their first two principal components. The top 500 genes, selected by highest row variance, were used to generate the plot. Additionally, differentially expressed genes between urticarial and control unaffected skin were identified using the package limma (3.48.3).^14^ The list of all genes, ranked according to fold change, was used to calculate enrichment of gene sets within Hallmark^15^ using package fgsea (1.18.0)^16^ with a random seed set to 42. 50 gene sets of interest were visualized using ggplot2 (3.3.5).^17^ R version 4.1.1 was used.

Supplementary Figures & Tables

**
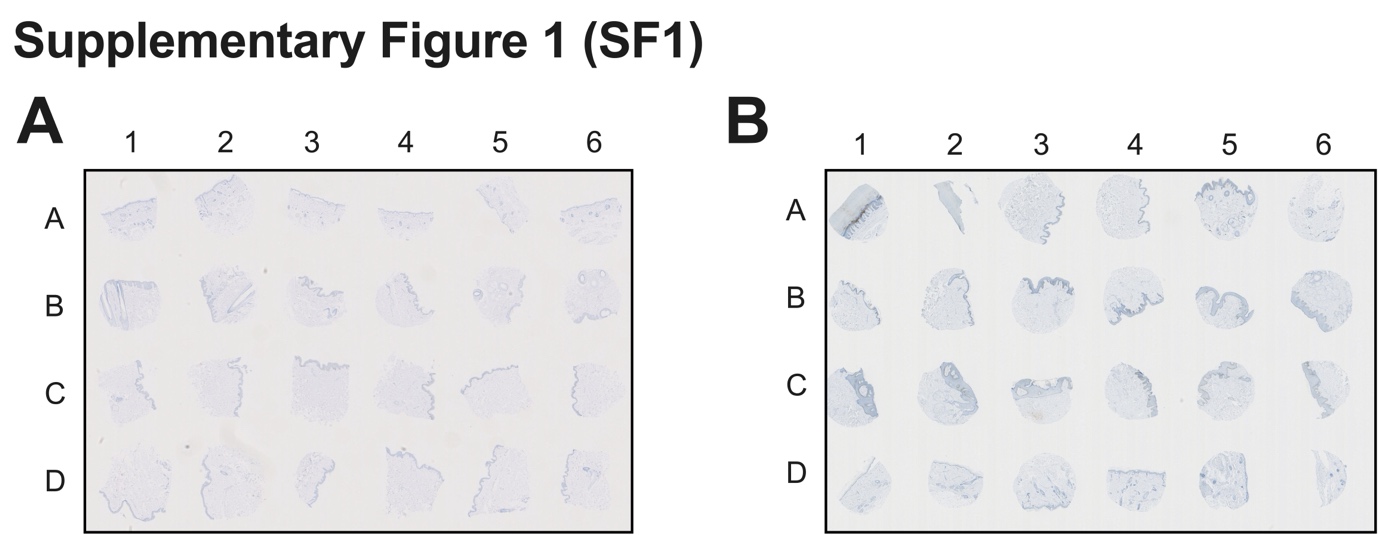
**

**Supplementary Figure 1 (SF1):** Key for normal skin tissue microarrays (TMA) positions shown in Figure 2A; TMA 1 (**A**) and TMA 2 (**B**). Full sample demographics information tables can be found in Supplementary Table 2 and 3, respectively.

**
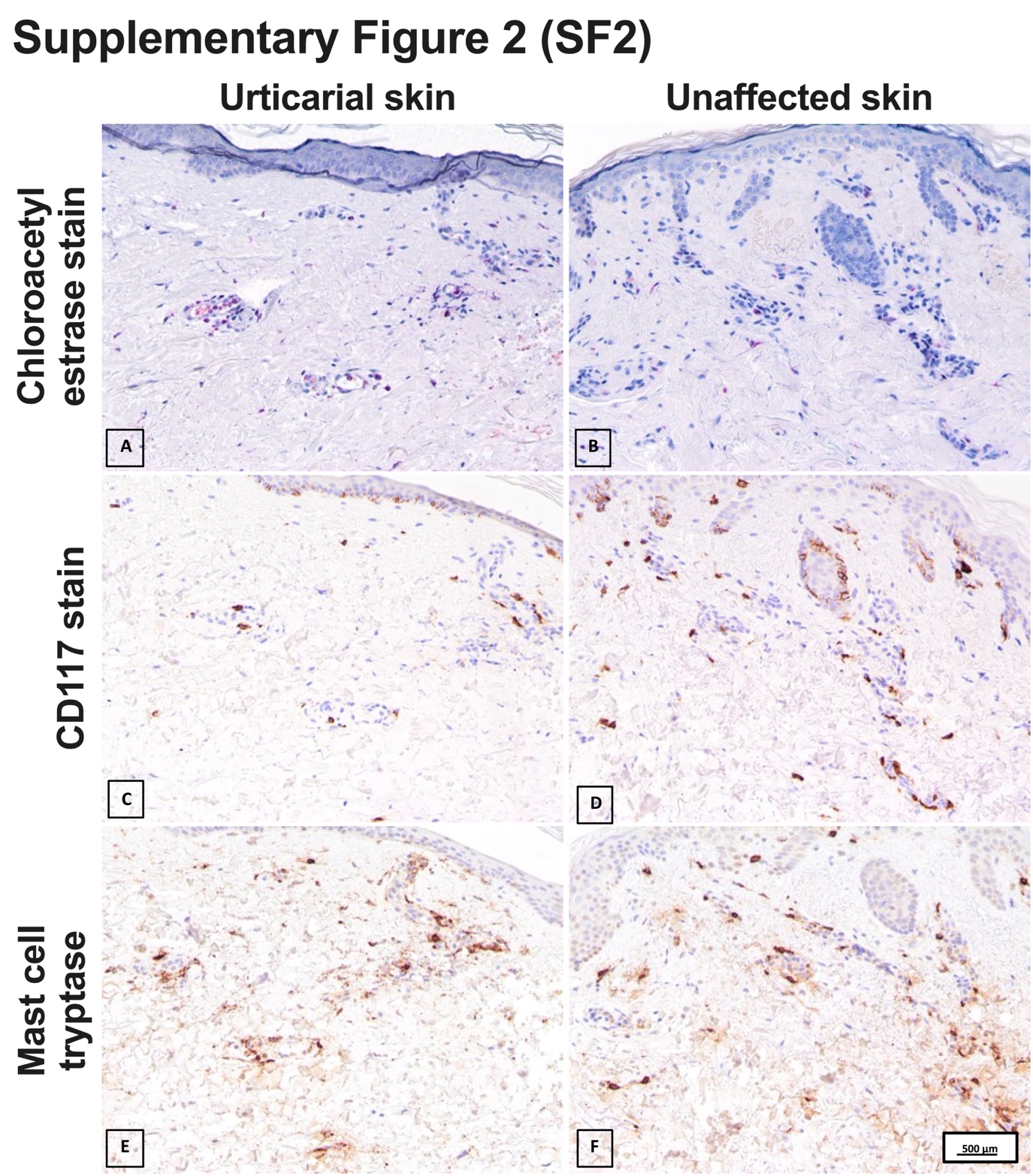
**

**Supplementary Figure 2 (SF2):** Chloroacetic esterase staining (CAE) of urticarial skin (A) and unaffected skin (B) of a patient treated with the highest dose levels of MOv18 IgE (Patient A). CD117 staining of urticarial skin (C) and unaffected skin (D). Mast cell tryptase (MCT) of urticarial skin (E) and unaffected skin (F). Magnification: 20x. Scale bar: 500µm.

**
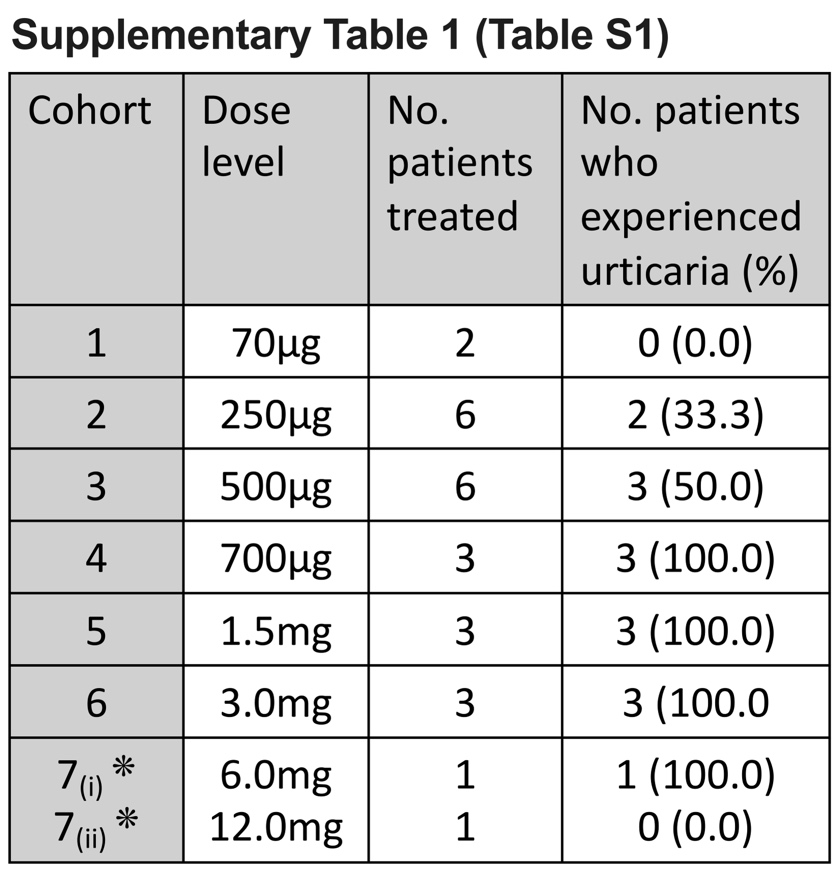
**

**Supplementary Table 1 (Table S1):** Table of cohort, dose, number of patients treated and number of those who experienced urticarial reactions in the Phase I clinical trial of MOv18 patients (*n*=24 treated, left).

**
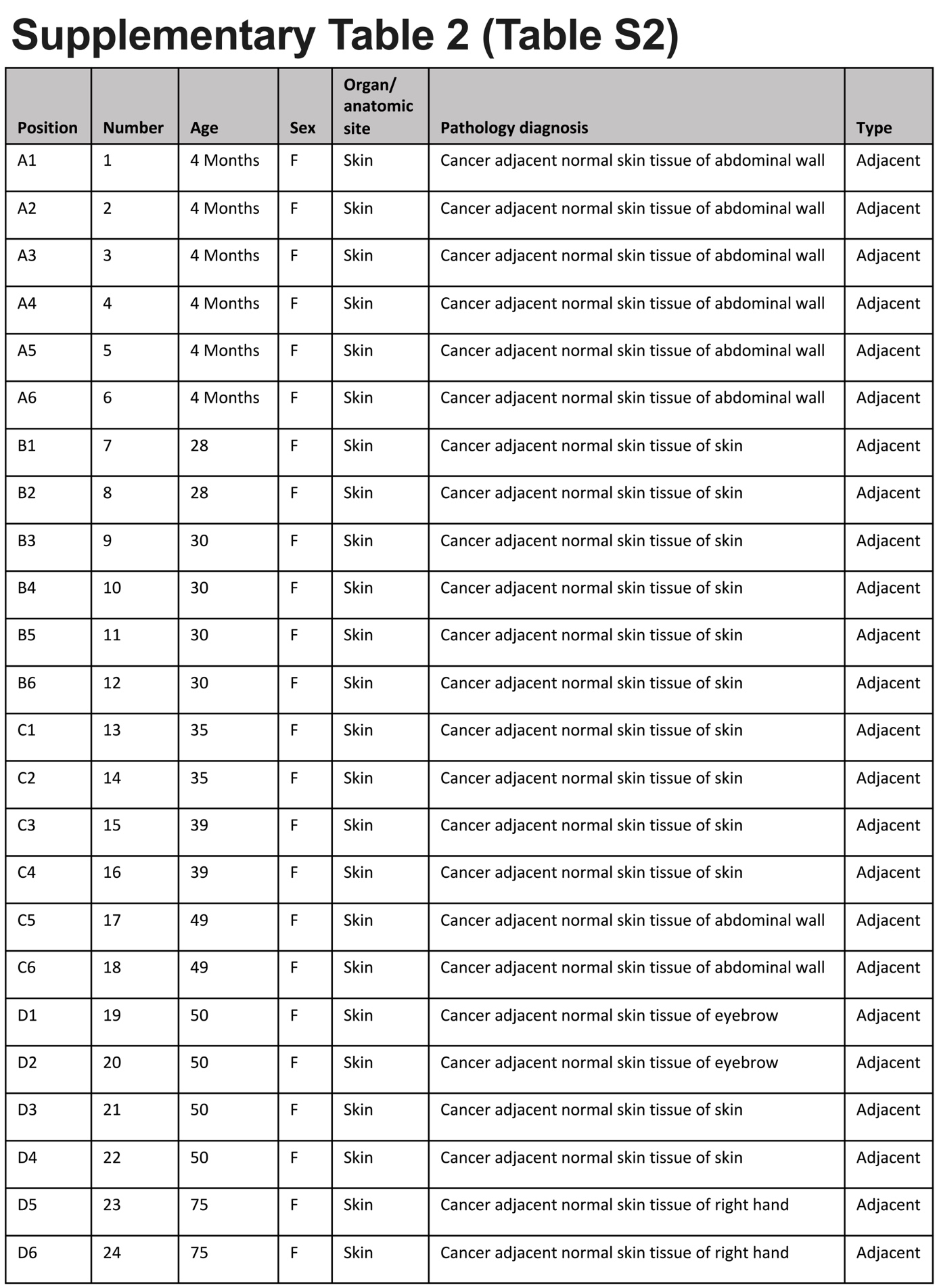
**

**Supplementary Table 2 (Table S2):** Table of sample demographic information for tissue microarray TMA 1. Key for positions of samples found in Supplementary Figure 1.

**
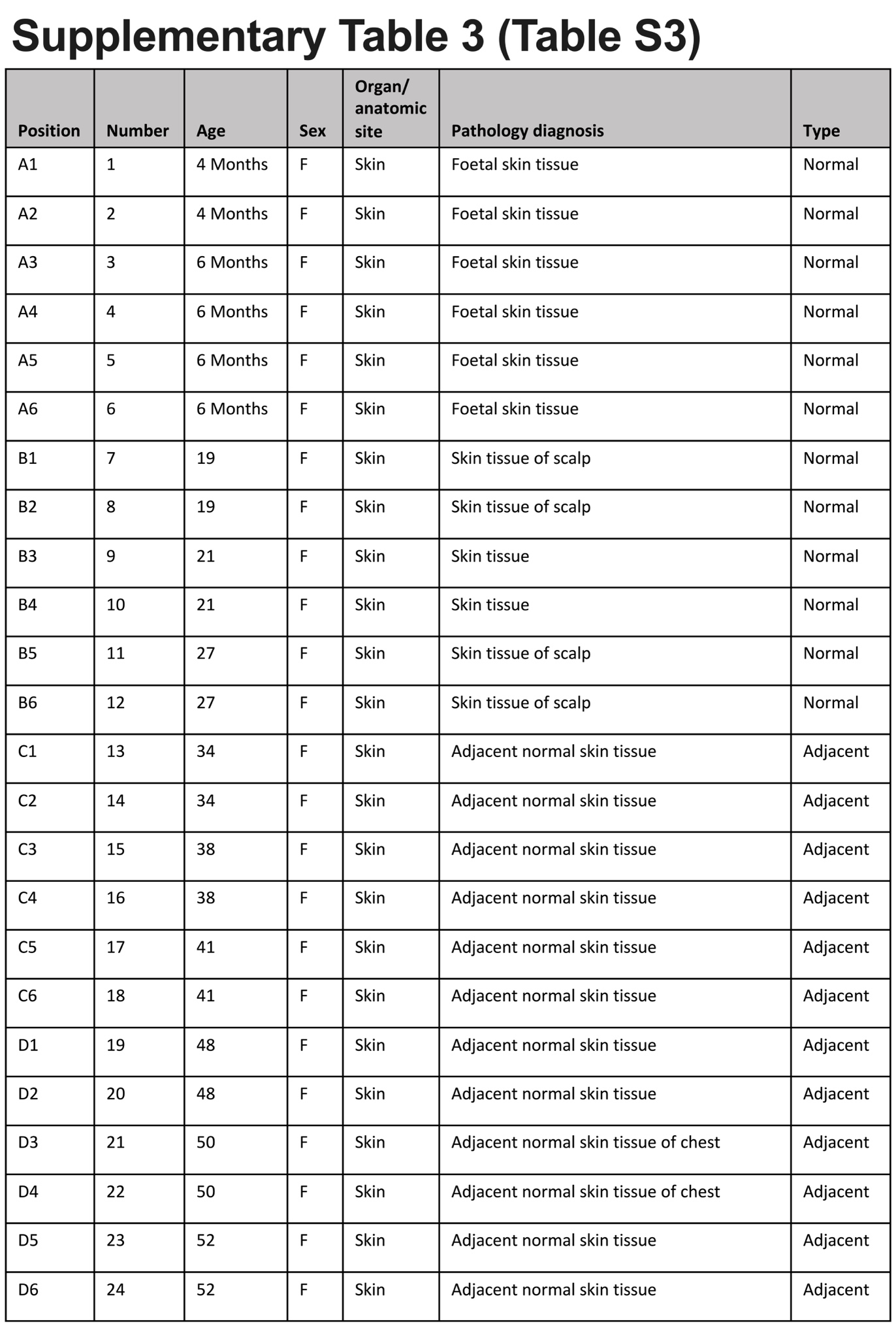
**

**Supplementary Table 3 (Table S3):** Table of sample demographic information for tissue microarray TMA 2. Key for positions of samples found in Supplementary Figure 1.


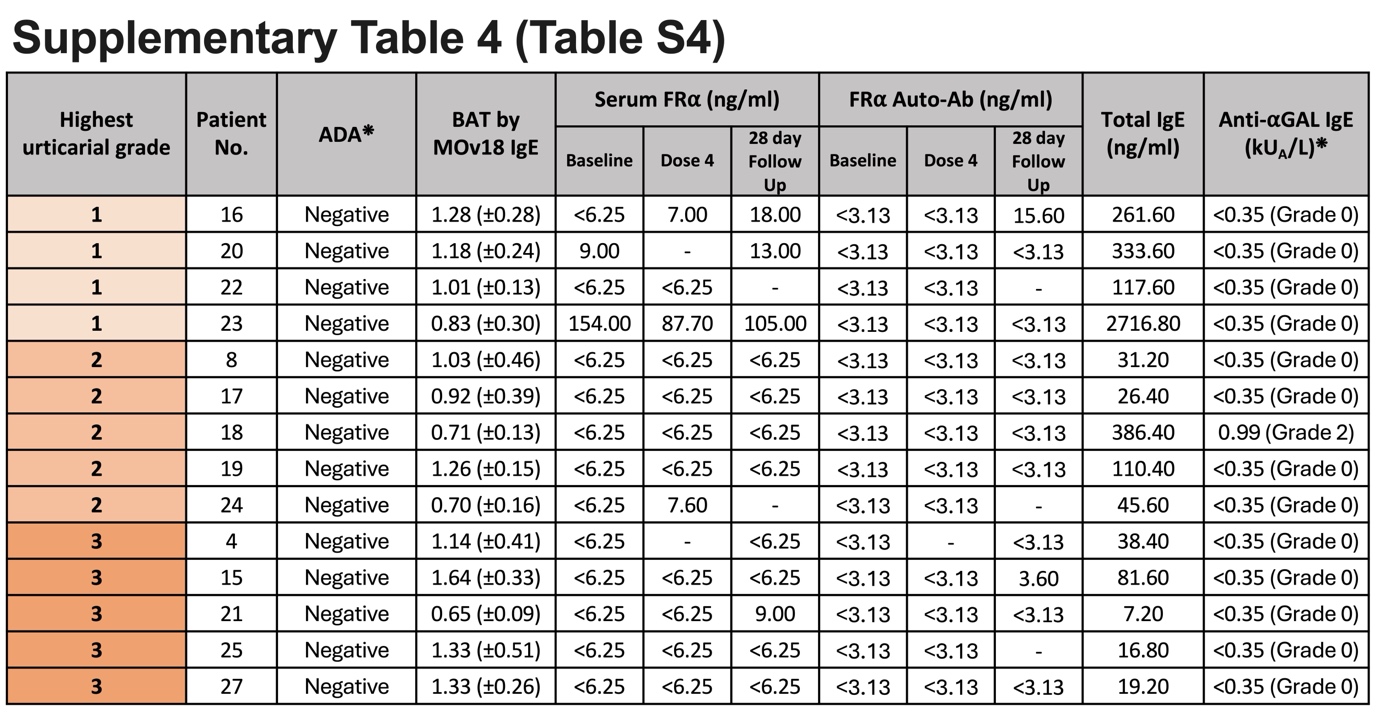


**Supplementary Table 4 (Table S4):** Circulatory markers and functional readouts of basophil activation in patients who experienced CTCAE urticaria without systemic anaphylactic reaction during treatment with MOv18 IgE. ^❋^ = measured at the earliest urticaria presentation. Basophil activation test (BAT) measured mean (±SEM) fold change in % CD63 expression by basophils, >3.00 fold cut off for positivity applied.

**
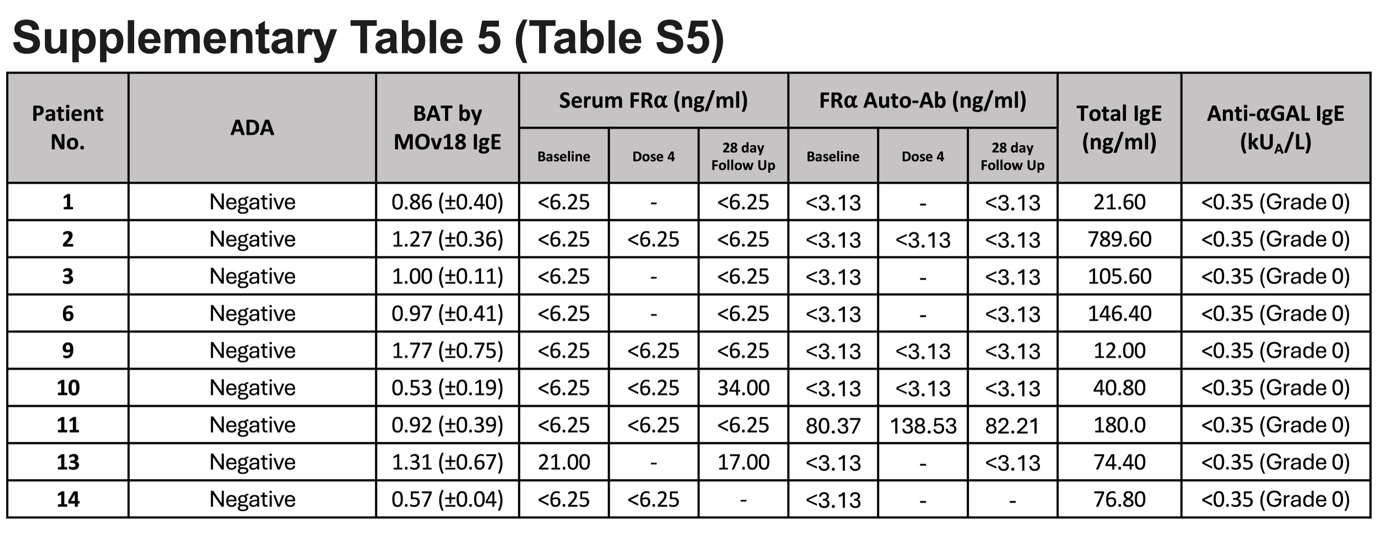
Supplementary Table 5 (Table S5):** Circulatory markers and functional readouts of basophil activation in patients who did not experience urticaria during treatment with MOv18 IgE. Anti-drug antibodies (ADA), and anti-⍺GAL IgE antibodies were measured at the earliest dose point. Basophil activation test (BAT) measured mean (±SEM) fold change in % CD63 expression of basophils, >3.00 fold cut off for positivity applied.


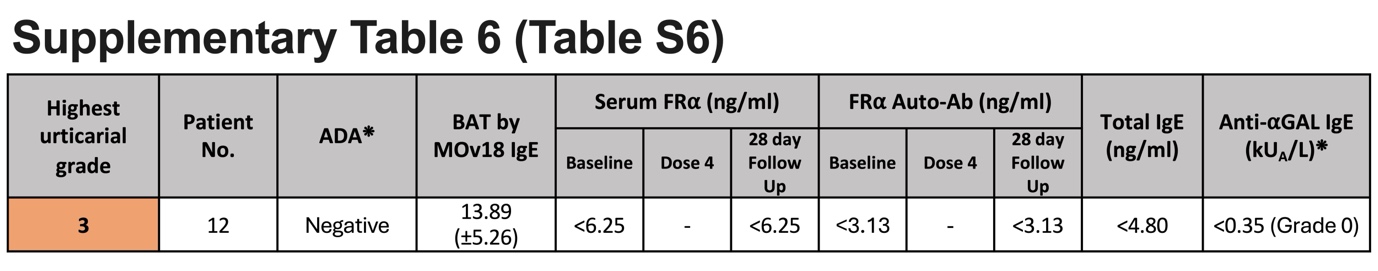


**Supplementary Table 6 (Table S6):** Circulatory markers and functional readouts of basophil activation in singular patient who experienced anaphylaxis during treatment with MOv18 IgE at their first dose. ^❋^ = measured at the earliest urticaria presentation. Basophil activation test (BAT) measured mean(±SEM) fold change in CD63 expression of basophils, >3.00 fold cut off for positivity applied.

**Supplementary References:**

1. Crescioli S, Correa I, Ng J, et al. B cell profiles, antibody repertoire and reactivity reveal dysregulated responses with autoimmune features in melanoma. *Nat Commun.* 2023;14(1):3378.

2. Di Meo A, Sohaei D, Batruch I, Alexandrou P, Prassas I, Diamandis EP. Proteomic Profiling of the Human Tissue and Biological Fluid Proteome. *J Proteome Res.* 2021;20(1):444-452.

3. Pellizzari G, Martinez O, Crescioli S, et al. Immunotherapy using IgE or CAR T cells for cancers expressing the tumor antigen SLC3A2. *J Immunother Cancer.* 2021;9(6).

4. Josephs DH, Bax HJ, Dodev T, et al. Anti-Folate Receptor-alpha IgE but not IgG Recruits Macrophages to Attack Tumors via TNFalpha/MCP-1 Signaling. *Cancer Res.* 2017;77(5):1127-1141.

5. Josephs DH, Nakamura M, Bax HJ, et al. An immunologically relevant rodent model demonstrates safety of therapy using a tumour-specific IgE. *Allergy.* 2018;73(12):2328-2341.

6. Nakamura M, Souri EA, Osborn G, et al. IgE Activates Monocytes from Cancer Patients to Acquire a Pro-Inflammatory Phenotype. *Cancers (Basel).* 2020;12(11).

7. Pellizzari G, Hoskin C, Crescioli S, et al. IgE re-programs alternatively-activated human macrophages towards pro-inflammatory anti-tumoural states. *EBioMedicine.* 2019;43:67-81.

8. Spicer J, Basu B, Montes A, et al. Safety and anti-tumour activity of the IgE antibody MOv18 in patients with advanced solid tumours expressing folate receptor-alpha: a phase I trial. *Nat Commun.* 2023;14(1):4180.

9. Passia E, Jandus P. Using Baseline and Peak Serum Tryptase Levels to Diagnose Anaphylaxis: a Review. *Clin Rev Allergy Immunol.* 2020;58(3):366-376.

10. Williams KW, Sharma HP. Anaphylaxis and urticaria. *Immunol Allergy Clin North Am.* 2015;35(1):199-219.

11. Rudman SM, Josephs DH, Cambrook H, et al. Harnessing engineered antibodies of the IgE class to combat malignancy: initial assessment of FcvarepsilonRI-mediated basophil activation by a tumour-specific IgE antibody to evaluate the risk of type I hypersensitivity. *Clin Exp Allergy.* 2011;41(10):1400-1413.

12. Bax HJ, Chauhan J, Stavraka C, et al. Basophils from Cancer Patients Respond to Immune Stimuli and Predict Clinical Outcome. *Cells.* 2020;9(7).

13. Bax HJ, Khiabany A, Stavraka C, et al. Basophil activation test in cancer patient blood evaluating potential hypersensitivity to an anti-tumor IgE therapeutic candidate. *Allergy.* 2020;75(8):2069-2073.

14. Ritchie ME, Phipson B, Wu D, et al. limma powers differential expression analyses for RNA-sequencing and microarray studies. *Nucleic Acids Res.* 2015;43(7):e47.

15. Liberzon A, Birger C, Thorvaldsdottir H, Ghandi M, Mesirov JP, Tamayo P. The Molecular Signatures Database (MSigDB) hallmark gene set collection. *Cell Syst.* 2015;1(6):417-425.

16. Korotkevich G, Sukhov V, Budin N, Shpak B, Artyomov MN, Sergushichev A. Fast gene set enrichment analysis. *bioRxiv.* 2021:060012.

17. Wickham H. *Ggplot2 : elegant graphics for data analysis.* Vol 1. 2 ed: Springer Cham; 2016.
